# Spontaneous unruptured unilateral twin tubal ectopic pregnancy: a case report and review of the literature

**DOI:** 10.3389/fmed.2026.1900321

**Published:** 2026-09-18

**Authors:** Xuan Zhou, Pin Li, Cuihua Wang, Min Kou, Huidi Wei

**Affiliations:** 1 Department of Diagnostic Ultrasound, Affiliated Hospital of Hebei University, Baoding, Hebei, China; 2 Department of Ultrasound, Baoding No.6 Hospital, Baoding, Hebei, China

**Keywords:** ectopic pregnancy, salpingectomy, spontaneous conception, tubal pregnancy, twin pregnancy

## Abstract

Ectopic pregnancy accounts for approximately 1%–2% of all pregnancy-related conditions. Among these cases, spontaneous unilateral twin tubal ectopic pregnancy is exceptionally uncommon, with an estimated incidence of approximately 1 in 125,000 spontaneous conceptions. We report a case of a 40-year-old woman with a history of three induced abortions who presented with 7 weeks of amenorrhea, 3 weeks + 2 days of intermittent vaginal spotting, and lower abdominal pain. Serial measurements of serum β-human chorionic gonadotropin (β-hCG) demonstrated a persistent and rapid increase, rising from 11,633.28 mIU/mL to 19,707.09 mIU/mL within four days. Transvaginal ultrasonography revealed two thick-walled cystic structures of increasing size within the left adnexal region, while no gestational sac was identified within the uterine cavity. Histopathological examination following diagnostic curettage showed decidualized endometrium with an Arias–Stella reaction but no chorionic villi. The patient subsequently underwent laparoscopic left salpingectomy. Postoperative pathological evaluation confirmed the presence of two distinct chorionic villous tissue masses, establishing the diagnosis of unilateral twin tubal pregnancy in the left fallopian tube. Our literature review indicated that most previously reported cases were associated with assisted reproductive technologies or identifiable risk factors for tubal damage, whereas spontaneous cases occurring in the absence of recognized predisposing factors remain exceedingly rare. This case highlights that twin ectopic pregnancy should be considered when serum β-hCG levels are disproportionately elevated, and no intrauterine gestational sac is visualized on ultrasonography, even in women with no apparent risk factors. Early recognition through serial ultrasonographic surveillance, in combination with timely surgical intervention, plays a critical role in preventing tubal rupture and optimizing clinical outcomes.

## Introduction

Ectopic pregnancy accounts for approximately 1%–2% of all pregnancy-related disorders (1). Clinically, ectopic pregnancies are classified by implantation site, with tubal gestation being the most common form (over 95%), followed by rarer variants including interstitial, cervical, cesarean scar, ovarian, abdominal, and heterotopic pregnancies (2). Twin ectopic pregnancy is an exceptionally rare clinical entity, with an estimated incidence of approximately 1 in 125,000 spontaneous conceptions (3). Furthermore, live twin ectopic pregnancies with detectable fetal cardiac activity are even more uncommon, representing a small subset of already rare cases (4). Compared with singleton ectopic pregnancy, twin ectopic pregnancy is generally associated with markedly higher serum human chorionic gonadotropin (hCG) concentrations and carries a substantially increased risk of severe complications, including tubal rupture and massive intraperitoneal hemorrhage, entailing significant threats to both future fertility and maternal safety (5).

Spontaneous unilateral twin tubal ectopic pregnancy is particularly uncommon. Existing literature consists predominantly of isolated case reports, many of which are associated with recognized risk factors, such as the use of assisted reproductive technologies, pelvic inflammatory disease, previous tubal surgery, or intrauterine contraceptive device use (6). In contrast to the more frequently reported non-viable or ruptured cases, unruptured live twin ectopic pregnancies diagnosed preoperatively remain exceedingly rare (7, 8). Nevertheless, sporadic cases have been reported in women without any identifiable predisposing factors. The pathogenesis in such patients remains poorly understood and may involve multiple mechanisms, including impaired tubal transport and abnormal embryonic cleavage (6). Given that clinical recognition of this condition is relatively low, and imaging findings may be confounded by the presence of pseudogestational sacs within the uterine cavity or corpus luteum cysts, there is a high risk of delayed diagnosis or misdiagnosis. Therefore, a better understanding of the clinical characteristics, ultrasonographic features, and effective management of this rare condition is essential for improving diagnostic accuracy and patient outcomes.

In this report, we describe a case of spontaneous unilateral unruptured twin tubal ectopic pregnancy in a 40-year-old woman. By reviewing the patient's clinical manifestations, serial ultrasonographic findings, and surgical pathology results, together with a review of the relevant literature, we aim to highlight the key diagnostic features and therapeutic considerations of this specific condition, thereby providing practical insights for clinical management.

## Case report

A 40-year-old woman (gravida 4, para 1) with a history of three induced abortions and no identifiable risk factors—including tubal surgery, pelvic inflammatory disease, intrauterine device use, or assisted reproductive technology—presented with 7 weeks of amenorrhea and 3 weeks of intermittent vaginal spotting. Table 1 summarizes the key demographic, clinical, laboratory, and imaging characteristics of this case.

**Table 1:** Summary of patient characteristics and clinical data.

Characteristic	Details
Demographics	
Age (years)	40
Gravida/Para	Gravida 4, Para 1
Previous pregnancies	Three induced abortions (dates unavailable)
Risk Factors	
Tubal surgery	None
Pelvic inflammatory disease/STI	None
Intrauterine device use	None
Assisted reproductive technology	None
Chronic medical conditions	None
Clinical Presentation	
Amenorrhea	49 days
Vaginal bleeding	Intermittent spotting for 23 days
Abdominal pain	Dull lower abdominal pain for 3 days
Associated symptoms	Intermittent rectal pressure
Vital Signs on Admission	
Blood pressure (mmHg)	115/70
Pulse rate (beats/min)	78
Body temperature ( °C)	36.5
Laboratory Findings	
Serum β-hCG on admission (mIU/mL)	11,633.28
Serum β-hCG on Day 5 (mIU/mL)	19,707.09
Hemoglobin (g/L)	124
White blood cell count (×10⁹/L)	6.8
Platelet count (×10⁹/L)	235
Transvaginal Ultrasonography	
Uterine cavity	Heterogeneous endometrium (16 mm), no gestational sac
Left adnexa (initial)	Two thick-walled cystic lesions: 11 × 10 × 9 mm and 8 × 8 × 7 mm
Left adnexa (follow-up, 3 days later)	Two thick-walled cystic lesions: 14 × 13 × 13 mm and 10 × 9 × 9 mm
Left ovary	Cystic lesion (20 × 15 × 15 mm), consistent with physiological cyst
Pelvic free fluid	None
Treatment	
Procedures	Diagnostic curettage followed by laparoscopic left salpingectomy
Intraoperative findings	Distended ampullary segment (∼4 cm), bluish-purple tense surface, no rupture
Pathology (curettage)	Secretory-phase endometrium with decidualization and Arias–Stella reaction; no chorionic villi
Pathology (salpingectomy)	Two distinct foci of chorionic villi and trophoblastic cells
Outcome	
Postoperative β-hCG (Day 2, mIU/mL)	3,457.94
Postoperative β-hCG (Day 4, mIU/mL)	1,728.62
Recovery	Uncomplicated, discharged on postoperative day 5

Phase 1: Initial presentation and referral (estimated gestational age ∼6 weeks). The patient had regular menstrual cycles, with her last menstrual period approximately 7 weeks before presentation. At approximately 3 weeks and 5 days after her missed period, she developed persistent, intermittent dark-red vaginal bleeding accompanied by occasional rectal pressure. Presuming this represented menstruation, she did not seek immediate care. After approximately 2 weeks of continued bleeding, she attended a local hospital. Ultrasonography revealed a small hypoechoic area within the uterine cavity (approximately 5 × 4 mm); serum hCG was measured, but the original laboratory report was unavailable. Vaginal bleeding persisted. Three days before admission to our hospital, she developed dull lower abdominal pain with worsening rectal pressure. Repeat ultrasonography showed a thickened, heterogeneous endometrium (14 mm), two cystic structures adjacent to the right ovary (17 × 16 mm and 13 × 13 mm), and a small amount of pelvic free fluid (maximum depth ∼13 mm). The serum hCG level was 11,633.28 mIU/mL, raising suspicion of ectopic pregnancy, and she was referred to our institution.

Phase 2: Admission evaluation (Day 1, estimated gestational age ∼7 weeks). On admission, vital signs were stable (blood pressure 115/70 mmHg, pulse 78 beats/min). Abdominal examination revealed mild lower abdominal tenderness without rebound tenderness or guarding. Gynecological examination showed a small amount of dark-red blood originating from the uterine cavity, a smooth cervix without motion tenderness, and an anteverted, soft uterus equivalent in size to a 7-week gestation. Mild tenderness was elicited in the left adnexal region, though no definite mass was palpable. Laboratory findings were unremarkable except for an elevated serum β-hCG of 11,633.28 mIU/mL. Transvaginal ultrasonography revealed a heterogeneous endometrium (16 mm) with no intrauterine gestational sac. Within the left ovary, a well-defined cystic lesion (20 × 15 × 15 mm) consistent with a physiological cyst was noted. Adjacent to the left ovary, two thick-walled cystic structures were identified (11 × 10 × 9 mm and 8 × 8 × 7 mm); neither a yolk sac nor an embryonic pole was visualized. No significant pelvic free fluid was detected.

Phase 3: Diagnostic interventions and clinical decision-making (Days 1–5). Based on the clinical presentation, rising β-hCG, absence of an intrauterine sac, and adnexal cystic lesions, a working diagnosis of ectopic pregnancy was made. Diagnostic curettage was performed to exclude intrauterine pregnancy. Histopathological examination showed secretory-phase endometrium with stromal decidualization and focal Arias–Stella reaction, but no chorionic villi or trophoblastic tissue (Figure 2), supporting an extrauterine gestation. Serial β-hCG measurements demonstrated a continuous rise: 14,434.18 mIU/mL on Day 3, 16,201.78 mIU/mL on Day 4, and 19,707.09 mIU/mL on Day 5. Follow-up ultrasonography on Day 4 showed progressive enlargement of the left adnexal lesions to 14 × 13 × 13 mm and 10 × 9 × 9 mm, with increased wall tension (Figure 1). These findings established the clinical diagnosis of unruptured left-sided twin tubal ectopic pregnancy. Given the continuous lesion enlargement and markedly elevated β-hCG levels, conservative management was considered unlikely to succeed. After detailed counseling, the patient elected surgical intervention and provided written informed consent. Figure 3 presents a simplified diagnostic flow diagram summarizing this chronological diagnostic pathway.

**Figure 1:**
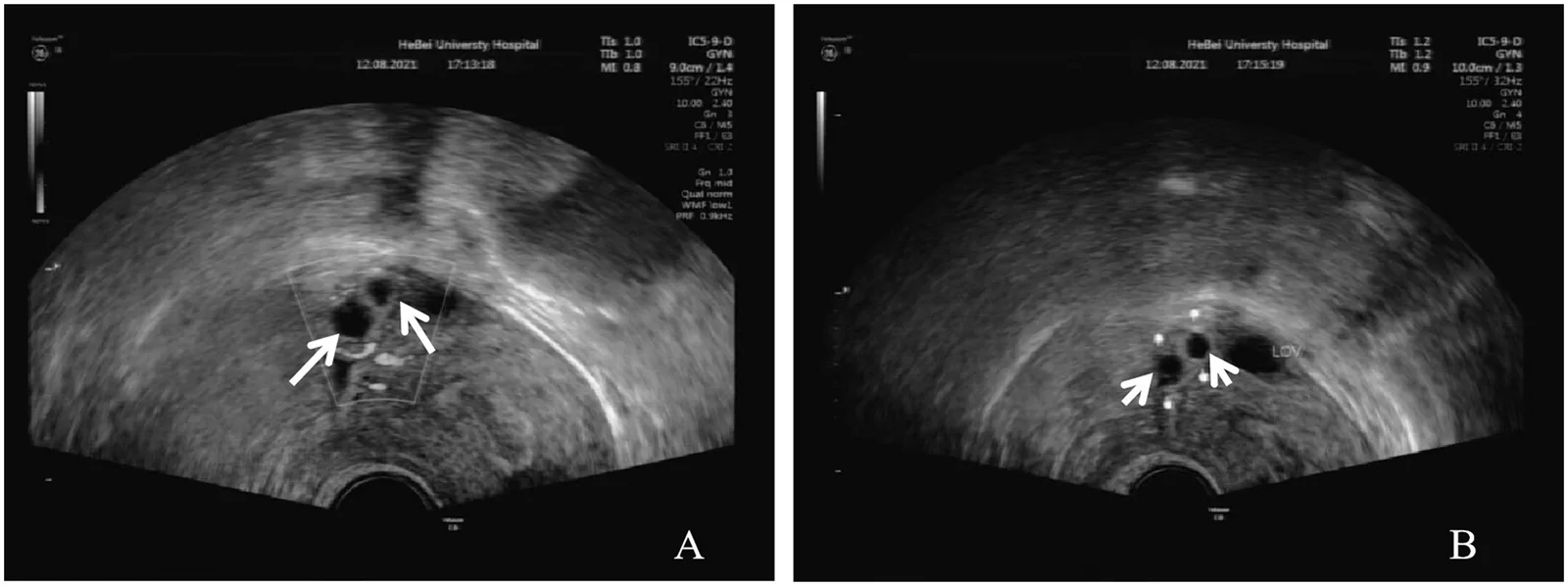
**(A,B)** Transvaginal ultrasonography demonstrating two thick-walled cystic lesions in the left adnexal region (indicated by arrows), suggestive of gestational sacs consistent with twin ectopic pregnancy.

**Figure 2:**
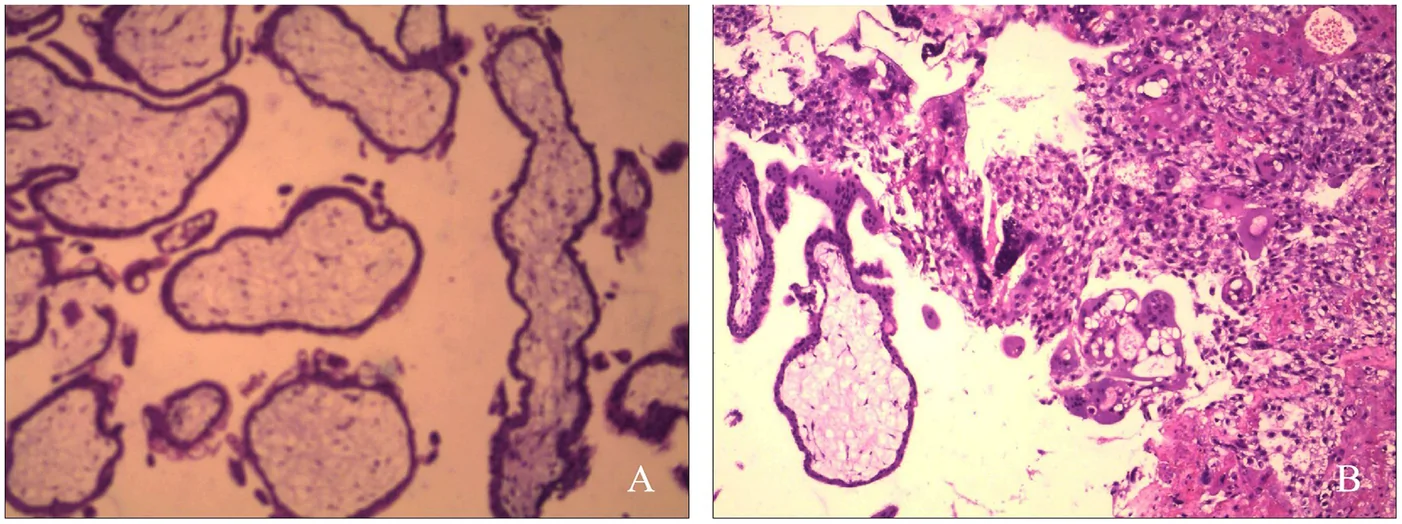
Histopathological findings confirming the diagnosis of twin tubal ectopic pregnancy. **(A)** Endometrial curettage specimen showing secretory-phase endometrium with stromal decidualization and focal Arias–Stella reaction (glandular nuclear atypia associated with ectopic pregnancy). No chorionic villi or trophoblastic cells are identified, excluding an intrauterine pregnancy (H&E, ×100). **(B)** Resected left fallopian tube specimen revealing two distinct foci of chorionic villi and trophoblastic cells, confirming twin ectopic gestation within the tubal lumen (H&E, ×100). The combined findings established the definitive diagnosis of unilateral twin tubal ectopic pregnancy.

**Figure 3:**
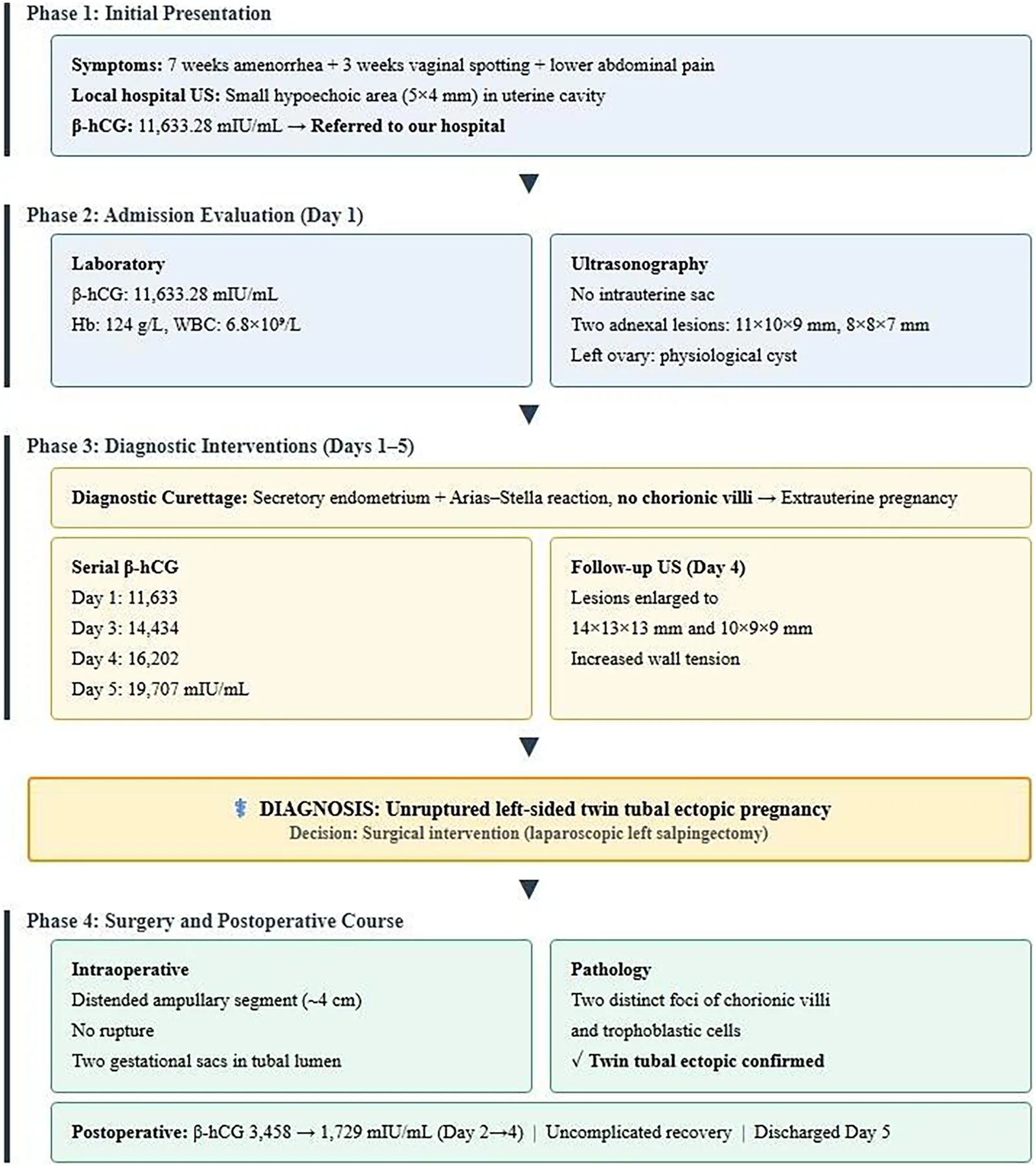
Simplified diagnostic flow diagram illustrating the chronological diagnostic pathway. Key steps include initial presentation, admission evaluation, diagnostic interventions (curettage, serial β-hCG, follow-up ultrasound), diagnosis, and surgical confirmation. The diagram highlights the integration of laboratory, imaging, and pathological findings that led to the preoperative diagnosis of unruptured left-sided twin tubal ectopic pregnancy.

Phase 4: Surgery and postoperative course (Day 5 onward, estimated gestational age ∼7 weeks + 5 days). Laparoscopic left salpingectomy was performed under general anesthesia. Intraoperatively, the ampullary segment of the left fallopian tube was markedly distended (approximately 4 cm in diameter), with a bluish-purple, tense surface, but no rupture was observed. The resected tube was opened longitudinally, revealing two separate and relatively intact gestational sacs within the tubal lumen. Histopathological examination confirmed two distinct foci of chorionic villi and trophoblastic cells (Figure 2), establishing the diagnosis of left-sided twin tubal ectopic pregnancy. Postoperatively, serum β-hCG declined to 3,457.94 mIU/mL on Day 2 and 1,728.62 mIU/mL on Day 4. The patient had an uncomplicated recovery and was discharged on Day 5. Weekly outpatient follow-up with serial β-hCG measurements was recommended until complete normalization. The diagnostic workflow is illustrated in Figure 3.

## Discussion

The present case highlights the diagnostic and therapeutic challenges posed by spontaneous unilateral twin tubal ectopic pregnancy. Our findings demonstrate that a high index of suspicion, prompted by disproportionately elevated serum β-hCG levels and the absence of an intrauterine gestational sac, is critical for early diagnosis. The successful preoperative identification of this unruptured case underscores the value of integrating serial ultrasonographic surveillance with histopathological correlation, enabling timely surgical intervention and favorable patient outcomes. This case is particularly instructive as it occurred in a woman without classical risk factors, reinforcing that this rare entity should be considered in a broader differential diagnosis.

The pathogenesis of twin tubal ectopic pregnancy is multifactorial, with established risk factors including pelvic inflammatory disease, prior tubal surgery, assisted reproductive technology (ART), and intrauterine device use (7). In the present case, the patient had a history of three induced abortions. Although this does not directly damage the fallopian tubes, repeated intrauterine instrumentation can lead to subclinical endometritis or alter the tubal microenvironment, potentially impairing ciliary function and embryo transport. Furthermore, the patient was of advanced maternal age (40 years). Age-related declines in tubal peristalsis and ciliary beat frequency may increase the residence time of the embryo within the tube, thereby elevating the risk of ectopic implantation (9). We acknowledge that immunological profiling and direct assessment of tubal ciliary function were not performed in this case, as such specialized investigations are not part of routine clinical practice in the acute management of ectopic pregnancy and are generally reserved for research settings. Similarly, sex hormone profiles (including estradiol, progesterone, and luteinizing hormone), ovarian reserve markers (anti-Müllerian hormone and follicle-stimulating hormone), and tubal patency testing were not obtained. These evaluations are not part of routine acute management of suspected ectopic pregnancy and do not influence emergent clinical decision-making. Their absence precludes any definitive conclusion regarding the pathophysiological mechanisms underlying this spontaneous twin ectopic pregnancy in an advanced-age patient, which we acknowledge as a limitation of this report. Future studies incorporating comprehensive endocrine and tubal function assessments may help clarify the pathogenesis of this rare entity. Consequently, the above pathogenetic interpretation remains speculative. Given the constraints of emergency clinical care, we have focused our discussion on the diagnostic and therapeutic aspects that are directly relevant to patient management, rather than on mechanistic exploration. However, it is important to note that the majority of reported cases are associated with ART, while spontaneous cases, like ours, remain exceptionally rare (6, 7). The absence of recognized risk factors in a subset of patients suggests the involvement of sporadic, unidentifiable factors or inherent embryonic abnormalities (6). Several mechanisms have been proposed for the development of twin ectopic pregnancy. One possibility is that two oocytes are released from the same ovary during a single ovulatory cycle and are fertilized independently, resulting in a dizygotic twin pregnancy. Alternatively, a single fertilized ovum may undergo cleavage during its transit through the fallopian tube, producing monozygotic twins that subsequently implant within the tubal mucosa. In this case, pathological examination demonstrated two distinct gestational sacs within the affected tube, but embryonic genotyping was not performed to determine zygosity. While genetic confirmation would be scientifically valuable, in routine clinical practice such analysis is rarely performed and does not influence the acute management of this emergent condition. From a clinical standpoint, distinguishing between monozygotic and dizygotic origins has no immediate bearing on treatment decisions, as the therapeutic approach—whether surgical or medical—is guided by the patient's hemodynamic status, β-hCG levels, ultrasonographic findings, and reproductive wishes, rather than by zygosity. We acknowledge this as a limitation of the study and recommend that future case reports consider genetic analysis when feasible to further elucidate the embryological pathways underlying this rare condition.

The cornerstone of ectopic pregnancy diagnosis is the combination of serum β-hCG measurements and transvaginal ultrasonography (TVS). A critical concept in this diagnostic algorithm is the discriminatory zone, the serum β-hCG level above which a normal intrauterine pregnancy (IUP) should be reliably visualized by TVS. While a commonly cited threshold is 1,500–2,500 mIU/mL, there is considerable inter- and intra-individual variability, and its reliability is limited (3). In this case, the initial β-hCG level on admission was 11,633.28 mIU/mL, far exceeding this threshold, yet no IUP was seen. This significant disparity strongly suggested an ectopic gestation. However, in the context of twin ectopic pregnancies, the β-hCG levels can be disproportionately high and may even overlap with those of normal twin IUPs (4). This overlap diminishes the utility of a single β-hCG value and the discriminatory zone, as reliance on this threshold could lead to a false sense of security or a delayed diagnosis. Our case illustrates that when β-hCG levels are markedly elevated in the absence of a corresponding intrauterine sac, especially when two adnexal structures are visible, the differential diagnosis must aggressively include a multiple ectopic pregnancy. Limitations of the discriminatory zone include the variability in ultrasound equipment, operator skill, and patient factors such as uterine fibroids or obesity. Therefore, while serial β-hCG trends are essential for monitoring, the diagnosis should be based on the integration of dynamic clinical, laboratory, and imaging findings rather than on a single numerical value.

Among the already rare phenomenon of twin tubal ectopic pregnancy, the presence of a live gestation, defined by detectable fetal cardiac activity, is an even rarer event (4). A systematic review by Sidhar et al. identified only a handful of such cases, most of which were associated with ART (5). For instance, Kim et al. and Trojnarska et al. reported cases where ultrasound clearly demonstrated two distinct gestational sacs with visible yolk sacs, embryonic poles, and even cardiac activity (3, 4). In contrast, our preoperative ultrasound examination, performed around 6–7 weeks of gestation, revealed only two thick-walled cystic structures without definitive visualization of embryonic structures or cardiac activity. This discrepancy may be explained by the relatively early gestational age at presentation or, more likely, by early embryonic arrest of both fetuses, a phenomenon previously suggested by Salloum et al. (7). It is plausible that the trophoblastic tissue responsible for the high β-hCG levels continues to proliferate even after embryonic demise, maintaining the ultrasonographic appearance of a cystic structure. This underscores that the absence of fetal cardiac activity on ultrasound does not exclude the diagnosis of twin ectopic pregnancy and should not be used to rule it out when other clinical and laboratory features are suspicious.

A key diagnostic pitfall is distinguishing an intrauterine pseudogestational sac from true gestational sacs. In our case, the referring hospital's ultrasound reported a small hypoechoic area in the uterine cavity, creating diagnostic uncertainty. Several features aid differentiation. A true intrauterine gestational sac is typically eccentrically located with a thick echogenic rim (double decidual sign) and contains a yolk sac or embryo by 5–6 weeks. A pseudogestational sac, in contrast, is centrally located, thin-walled, irregular, empty, and shows no growth on serial scans. For twin ectopic pregnancies, the presence of two thick-walled adnexal cystic structures—with progressive enlargement on follow-up ultrasound—provides the critical diagnostic clue. Importantly, the absence of a yolk sac or embryo does not exclude ectopic gestation, as trophoblastic tissue may continue to proliferate after embryonic arrest, as observed in our case. When uncertainty persists, diagnostic curettage remains valuable to confirm absence of chorionic villi and establish the extrauterine nature of the pregnancy.

In our patient, diagnostic uterine curettage was performed to definitively exclude an intrauterine pregnancy before proceeding to surgery. Its primary rationale was to confirm the absence of chorionic villi, which, in the context of markedly elevated β-hCG and adnexal masses, would strongly support the diagnosis of an ectopic gestation. This step was particularly critical given the initial ultrasound report from the referring hospital, which noted a small hypoechoic area in the uterine cavity, raising the potential for a pseudogestational sac or a very early IUP. In contemporary practice, the necessity of curettage in every suspected ectopic pregnancy is debated, as management increasingly relies on the more conservative “expectant” or “medical” approaches guided by serial β-hCG and ultrasound. For many patients, curettage can be avoided if a definitive adnexal mass containing a yolk sac or embryo is identified on ultrasound. However, in cases like ours where the adnexal findings are equivocal (i.e., thick-walled cysts without clear embryonic structures), curettage plays a crucial role in differentiating an early failing IUP from an ectopic pregnancy (10). It is indicated when the ultrasound is non-diagnostic and the β-hCG level is above the discriminatory zone, or when there is a clinical suspicion of an abnormal IUP (11). The benefits of curettage include its ability to provide a rapid and definitive pathological diagnosis, thereby preventing unnecessary surgical exploration for an IUP. The risks are minimal but include uterine perforation, infection, and the theoretical risk of disrupting an early IUP. In this case, the pathological finding of a decidualized endometrium with an Arias–Stella reaction and no chorionic villi was pivotal in confirming the extrauterine location of the pregnancy, justifying the subsequent decision for laparoscopic surgery. Recent reports have similarly underscored the critical role of integrating ultrasound and histopathological findings in the accurate diagnosis of twin ectopic pregnancies, supporting the diagnostic algorithm employed in our case (12).

Currently, no standardized guidelines exist specifically for unilateral twin tubal ectopic pregnancy; management is extrapolated from protocols for singleton ectopic pregnancies. Treatment options include medical (methotrexate), surgical, and expectant management. However, for twin ectopic pregnancies, medical management is particularly problematic. Methotrexate (MTX), a folic acid antagonist, is a first-line treatment for unruptured, hemodynamically stable ectopic pregnancies. Its advantages are its non-invasive nature and preservation of tubal integrity. However, its use in twin ectopic pregnancies has been fraught with a high failure rate. The greater trophoblastic tissue burden in twin pregnancies results in disproportionately elevated β-hCG levels, making MTX monotherapy less effective (5, 13). The criteria for successful MTX therapy—such as a β-hCG level below 5,000 mIU/mL, absence of fetal cardiac activity, and small ectopic mass—are often not met in twin cases (13). For instance, Betti et al. reported a case that required surgery after three courses of multi-dose MTX due to lesion enlargement and intraperitoneal hemorrhage (13). Furthermore, the use of MTX carries known complications, including severe abdominal pain, bone marrow suppression, and, rarely, pulmonary toxicity (14, 15). When medical therapy fails, as frequently observed in twin pregnancies, emergent surgical intervention for tubal rupture or hemorrhage becomes more complex and carries higher risks for the patient (15). Therefore, medical therapy is generally not recommended as a primary treatment for “pure” twin tubal ectopic pregnancies unless the patient is highly selected with low and declining β-hCG levels, which is an exceedingly rare scenario.

Surgery, particularly via the laparoscopic approach, remains the gold standard for twin tubal ectopic pregnancy. The choice between salpingectomy (complete removal of the tube) and salpingostomy (incision and removal of the pregnancy with tube preservation) is guided by the patient's hemodynamic stability, the extent of tubal damage, and the patient's desire for future fertility. Salpingectomy is the definitive treatment for tubal rupture or in hemodynamically unstable patients. It effectively eliminates the risk of persistent ectopic pregnancy and ipsilateral recurrence (16, 17). A meta-analysis by Ozcan et al. concluded that while salpingectomy does not significantly differ from salpingostomy in terms of future intrauterine pregnancy rates, it is associated with a lower risk of recurrent ectopic pregnancy (17). Postoperative hysteroscopic tubal flushing can effectively shorten the time to subsequent intrauterine pregnancy after unilateral salpingectomy for tubal ectopic pregnancy, though it cannot reduce the incidence of recurrent ectopic pregnancy (18). This approach was chosen for our 40-year-old patient who had completed her family, providing a definitive cure with minimal risk. Salpingostomy is considered for patients with a strong desire to preserve fertility, particularly in the setting of a contralateral tubal abnormality. It aims to preserve tubal function. However, its disadvantages include a higher risk of persistent trophoblastic disease (requiring MTX follow-up), a 10%–15% rate of ipsilateral recurrence, and the possibility of tubal damage that still compromises future fertility (17). Laparoscopic surgery offers the advantages of minimal invasiveness, shorter hospital stay, and faster postoperative recovery compared to open surgery. Open surgery is typically reserved for cases with hemodynamic instability, extensive intra-abdominal adhesions, or where laparoscopic expertise is unavailable.

In the management of this rare condition, and especially in the choice between radical and conservative surgery, patient counseling is paramount. The patient's age, history of infertility, and future reproductive plans must be central to the decision-making process. For a young patient with a strong desire for future children, a salpingostomy, with its attendant risks and need for close surveillance, might be considered a viable option. In contrast, for an older patient with completed family, like ours, a salpingectomy provides the safest and most definitive resolution. The surgeon must clearly communicate the success rates, risks, and fertility implications of each approach to enable the patient to make an informed, autonomous decision. This is particularly critical in twin ectopic pregnancies, where the stakes are higher due to the increased risk of medical treatment failure and surgical complications.

A systematic review of the literature shows that since the first report by De Ott in 1891, the total number of documented tubal twin ectopic pregnancies worldwide has remained limited, and cases occurring after spontaneous conception, particularly unruptured unilateral presentations, are exceedingly rare (1, 19). Most published reports consist of isolated case descriptions or small case series, and high-quality evidence from large prospective studies or randomized controlled trials is lacking. To provide a structured answer to the core clinical question—how to recognize and manage this rare condition—we synthesized the available literature together with our case, focusing on three practical aspects: diagnostic clues, imaging differentiation, and treatment selection. From a diagnostic standpoint, several recurring features emerge across the limited case series. The earliest and most consistent warning sign is disproportionately elevated serum β-hCG—frequently far exceeding the discriminatory zone in the absence of a corresponding intrauterine gestational sac—which was observed in nearly all reported cases, including ours. Second, transvaginal ultrasonography consistently revealed two adnexal cystic structures, even when definitive embryonic structures or cardiac activity were not yet visible; this “twin cystic sign” should therefore raise prompt suspicion for twin ectopic gestation. Third, in diagnostically equivocal presentations, diagnostic curettage played a pivotal role: pathological demonstration of a decidualized endometrium with Arias–Stella reaction in the absence of chorionic villi effectively excluded intrauterine pregnancy and provided critical confirmatory evidence, as exemplified in our case.

Regarding therapeutic strategy, analysis of treatment outcomes across reported cases reveals a notable pattern: methotrexate (MTX) monotherapy is associated with a substantially higher failure rate in twin ectopic pregnancies compared with singleton ectopic pregnancies, largely attributable to the greater trophoblastic tissue burden and correspondingly higher β-hCG levels. Most cases initially managed medically ultimately required surgical rescue, often under more urgent circumstances. Consequently, surgical intervention—particularly laparoscopic salpingectomy—emerges as the preferred first-line approach in hemodynamically stable patients who have completed their family planning. For patients with a strong desire for future fertility, salpingostomy may be considered after thorough counseling regarding the increased risks of persistent ectopic pregnancy and ipsilateral recurrence. Pragmatically, we suggest that β-hCG levels exceeding 5,000 mIU/mL or detectable fetal cardiac activity in a suspected twin tubal ectopic pregnancy should strongly favor surgical over medical management.

These synthesized observations, while necessarily preliminary given the rarity of the condition, provide a practical reference framework that may assist clinicians in navigating this uncommon but clinically significant entity.

A formal case–control comparison is not feasible within a single case report. Nonetheless, qualitative comparisons with literature-documented features of singleton tubal ectopic pregnancies offer useful insights. Biochemically, while singleton tubal ectopic pregnancies typically present with β-hCG levels near or below the discriminatory zone (1,500–2,500 mIU/mL), our patient exhibited markedly elevated levels (11,633–19,707 mIU/mL), reflecting the greater trophoblastic mass of twin gestation. Ultrasonographically, the presence of two distinct adnexal cystic structures—the “twin cystic sign”—contrasts with the single adnexal mass typically seen in singleton cases. Therapeutically, MTX failure rates appear higher in twin ectopic pregnancies; thus, when β-hCG exceeds 5,000 mIU/mL or fetal cardiac activity is detected, primary surgical management is more strongly favored than in singleton cases. We acknowledge the inherent limitations of single-case observations and emphasize that multi-center registries are needed for robust comparative analyses.

Given the absence of established guidelines specifically for this rare entity, we propose the following preliminary framework based on our case experience and literature synthesis. For β-hCG monitoring, we suggest serial measurements at 48-hour intervals during the diagnostic phase, as this frequency allows reliable assessment of rising trends while avoiding unnecessary delays; in twin ectopic pregnancies, the discriminatory zone threshold of 1,500–2,500 mIU/mL should be interpreted with particular caution given the disproportionately elevated levels typically observed. For ultrasound surveillance, we recommend repeat transvaginal ultrasonography within 48–72 h when initial findings are equivocal (e.g., adnexal cystic structures without definitive embryonic structures), as progressive enlargement of these lesions—as demonstrated in our case—provides a critical diagnostic clue. Regarding MTX therapy, we advocate against routine use of the standard single-dose protocol (50 mg/m^2^) in twin ectopic pregnancies when β-hCG exceeds 5,000 mIU/mL or fetal cardiac activity is present, given the substantially higher failure rates reported in the literature. In such patients, primary surgical management should be strongly preferred. These suggestions, while preliminary and requiring validation through larger case series, offer a practical reference for clinicians encountering this uncommon condition.

We acknowledge that long-term postoperative follow-up data are incomplete in this case. Serum β-hCG was monitored until postoperative Day 4 (declining to 1,728.62 mIU/mL), after which the patient was discharged and advised to attend weekly outpatient follow-ups until complete seroconversion. However, she did not return for subsequent measurements, and the exact time to β-hCG normalization, menstrual recovery, and long-term reproductive outcomes were not documented. This limitation reflects both the patient's completion of family planning—rendering future fertility less relevant—and the practical challenges of sustained follow-up in a case report context. We emphasize the importance of comprehensive longitudinal surveillance in future cases to better characterize the complete postoperative course of this rare condition.

## Conclusion

The clinical significance of these rare cases is twofold. First, they remind clinicians that even in the absence of obvious risk factors, discordance between serum β-hCG levels and ultrasonographic findings should prompt careful consideration of ectopic pregnancy, with particular attention paid to the possibility of twin or multiple ectopic gestations. Second, the successful management in the present case suggests that standardized serial ultrasonographic monitoring, a cautious and systematic diagnostic approach, and timely surgical intervention can significantly improve clinical outcomes and prevent severe complications such as tubal rupture and hemorrhagic shock. Future research should focus on accumulating larger case datasets and establishing multicenter registries in order to better characterize this rare condition, refine individualized treatment strategies, and ultimately develop consensus-based guidelines for the clinical management of twin ectopic pregnancy.

## Data Availability

The raw data supporting the conclusions of this article will be made available by the authors, without undue reservation.
